# Machine Learning in Clinical Psychology and Psychotherapy Education: A Mixed Methods Pilot Survey of Postgraduate Students at a Swiss University

**DOI:** 10.3389/fpubh.2021.623088

**Published:** 2021-04-09

**Authors:** Charlotte Blease, Anna Kharko, Marco Annoni, Jens Gaab, Cosima Locher

**Affiliations:** ^1^General Medicine and Primary Care, Beth Israel Deaconess Medical Center, Harvard Medical School, Boston, MA, United States; ^2^Faculty of Health, University of Plymouth, Plymouth, United Kingdom; ^3^Interdepartmental Center for Research Ethics and Integrity CNR, Rome, Italy; ^4^Fondazione Umberto Veronesi, Milan, Italy; ^5^Department of Clinical Psychology and Psychotherapy, University of Basel, Basel, Switzerland; ^6^Department of Consultation-Liaison Psychiatry and Psychosomatic Medicine, University Hospital Zurich, Zurich, Switzerland

**Keywords:** artificial intelligence, machine learning, psychology students, attitudes, opinions, survey, ethics, medical education psychotherapy education

## Abstract

**Background:** There is increasing use of psychotherapy apps in mental health care.

**Objective:** This mixed methods pilot study aimed to explore postgraduate clinical psychology students' familiarity and formal exposure to topics related to artificial intelligence and machine learning (AI/ML) during their studies.

**Methods:** In April-June 2020, we conducted a mixed-methods online survey using a convenience sample of 120 clinical psychology students enrolled in a two-year Masters' program at a Swiss University.

**Results:** In total 37 students responded (response rate: 37/120, 31%). Among respondents, 73% (*n* = 27) intended to enter a mental health profession, and 97% reported that they had heard of the term “machine learning.” Students estimated 0.52% of their program would be spent on AI/ML education. Around half (46%) reported that they intended to learn about AI/ML as it pertained to mental health care. On 5-point Likert scale, students “moderately agreed” (median = 4) that AI/M should be part of clinical psychology/psychotherapy education. Qualitative analysis of students' comments resulted in four major themes on the impact of AI/ML on mental healthcare: (1) Changes in the quality and understanding of psychotherapy care; (2) Impact on patient-therapist interactions; (3) Impact on the psychotherapy profession; (4) Data management and ethical issues.

**Conclusions:** This pilot study found that postgraduate clinical psychology students held a wide range of opinions but had limited formal education on how AI/ML-enabled tools might impact psychotherapy. The survey raises questions about how curricula could be enhanced to educate clinical psychology/psychotherapy trainees about the scope of AI/ML in mental healthcare.

## Introduction

### Background

Digital services based on artificial intelligence and machine learning (AI/ML) are increasingly used in mental health care including the use of apps. Health apps encompass a range of proposed uses, including the monitoring and tracking of symptoms, as well as direct-to-consumer interventions designed to support, complement, or replace, psychotherapy (1, 2). Psychotherapy apps have been designed to include various techniques including cognitive behavioral therapy, acceptance commitment therapy, and eclectic therapy. The recent coronavirus crisis has further accelerated the shift toward a model in which therapeutic relationships are increasingly mediated by on-line platforms and digital services.

Considering these digital advances, educating future clinicians, including psychologists and psychotherapists, will be important to ensure optimal, safe use of AI/ML enabled tools and innovations. So far, a growing number of investigations have explored the views of clinicians including primary care physicians on the impact of AI/ML tools on their job (3–7). These studies, albeit limited, suggest that mental health clinicians expect AI/ML to influence or change their professional roles in the future. For example, in 2020, an international survey of 791 psychiatrists reported that 75% (*n* = 593) believed that AI/ML enabled tools would, at some point, be able to fully replace psychiatrists in documenting and updating clinical records (7). In the same survey, 54% (*n* = 427) of psychiatrists believed that AI/ML tools will be able to fully replace humans in synthesizing information to make diagnoses. In qualitative research, psychiatrists express divergent opinions on the benefits and harms of AI/ML in treating mental health patients with comments demonstrating scarce reflection of ethical and regulatory considerations for patient care (6). Similarly, in a recent survey of psychiatrists in France (*n* = 515) (8), respondents expressed “moderate acceptability” of disruptive technologies, such as wrist bands for monitoring symptoms, but concluded that this likely reflected lack of extensive knowledge about these technologies.

## Objectives

In this study, our aim was to explore the opinions, openness, and familiarity of clinical psychology students on the impact of AI/ML on their job. In January 2020 we performed a scoping review of the literature using the terms “artificial intelligence,” “psychotherapy,” “education,” and “training” in the search engines PubMed, PsychInfo, and Google Scholar. This revealed very limited research examining attitudes toward artificial intelligence among students. So far, only one study has explored the awareness, and formal education of medical students about AI (9). Our objective was to initiate research into psychotherapy and clinical psychology education by launching a pilot survey of students. Specifically, we aimed to explore whether clinical psychology students believed their career choice would be impacted by AI/ML, the benefits, and harms of any such impact, and their level of formal training on these topics. Using a convenience sample of clinical psychology students at a leading European University, we aimed to investigate whether more education may be required so that trainee clinical psychologists/psychotherapists might ethically harness and advise patients about AI/ML-enabled tools.

## Methods

### Study Population

The single-center study was based at the Faculty of Psychology, University of Basel, Switzerland. The online survey was conducted from April to June 2020 with clinical psychology students (see Supplementary File 1). Students were 1st- and 2nd-year postgraduate students enrolled on a 2-year Masters' degree program in clinical psychology and psychotherapy (https://psychologie.unibas.ch/en/studies/master-program/).

Respondents enrolled in the Masters' program were invited via email to participate in the study. Three further reminder emails were sent, 1–2 weeks apart. Participation was voluntary and students were advised that the survey was not a test, that their responses would be pseudonymized, and that no sensitive information would be collected. There was no selection or exclusion in recruitment, and no reimbursement or compensation. Ethical approval for the study was granted by the Faculty of Psychology, University of Basel. The survey was administered in English, as students enrolled on the clinical psychology/psychotherapy Masters' program at the University of Basel are expected to be fluent English-speakers.

### Survey Instrument

The online survey [see Supplementary File 1] was designed with the online software Jisc (https://www.jisc.ac.uk/). The survey instrument was devised with consultation from academic informaticians at Harvard Medical School, and with psychotherapists at the University of Basel the survey was pre-tested with psychology students from outside the University to ensure face validity and feasibility. The survey opened with a brief statement: “We are inviting you, as psychology students to give your opinions about technology and the future of mental health care.” We also made it clear that the survey was aimed at assessing their personal opinions. We stated that we did not assume that participants have any expertise about AI/ML.

In the first section, respondents were asked to provide demographic information. Participants were also requested to state whether they intend to enter a mental health profession or not. The second section consisted of open comment questions on the future of psychotherapy (see Table 1, and Supplementary File 1). Respondents were requested to briefly describe way(s) in which AI/ML might change the care of patients with mental health conditions and psychotherapists' job in the next 25 years, as well as potential benefits and risks of AI/ML in the care of patients with mental health problems. The third section of the survey was intended to gauge participants' familiarity with artificial intelligence and machine learning. Participants were asked whether they were familiar with “machine learning” and “big data analytics” and whether they had read any academic journal articles relating to these topics (“no”, “yes” answers). Students were also requested to estimate the amount time (a) already spent and (b) anticipated on these topics in their program of study. Finally, respondents were also asked to rate the importance of AI/ML for clinical psychology/psychotherapy education.

**Table 1:** Open-ended questions.

In the next 25 years, please briefly describe the way(s) you believe artificial intelligence/machine learning might change the care of patients with mental health conditions.
In the next 25 years, please briefly describe the way(s) you believe artificial intelligence/machine learning might change the job of clinical psychologists and psychotherapists.
Please provide any brief comments you may have about the *potential benefits* of artificial intelligence/machine learning to the care of patients with mental health conditions.
Please provide any brief comments you may have about the *potential harms* of artificial intelligence/machine learning in the care of patients with mental health conditions.

### Data Management and Analysis

We used descriptive statistics to examine students' characteristics and opinions about the impact of AI/ML on the future of psychotherapy. The quantitative survey data was analyzed to extract summary statistics and 95% confidence intervals. Spearman's correlation coefficient was calculated for key variables describing students' experiences and attitudes toward including education about AI/ML in a clinical psychology program.

Survey responses were uploaded to the software QCAmap (coUnity Software Development GmbH) for analysis. Thematic content analysis was used to investigate students' responses. Transcripts were read several times by the two main coders (MA and CL) to achieve familiarization with the responses. Next, a process was employed in which brief descriptive labels (“code”) were applied to comments by two main coders (MA and CL); multiple codes were applied if quotations presented multiple meanings. Comments and codes were reviewed alongside an independent coder (AK), and further revisions and refinements of codes were undertaken until consensus was reached. Afterward, first-order codes were grouped into second-order themes based on commonality of meaning. All authors met to review and refined the final themes.

## Results

### Respondent Characteristics

Descriptive statistics and analysis were carried out using JASP (0.9.2). Table 2 provides a summary of demographic characteristics. The final respondent sample comprised 37 students (response rate: 37/120, 31%). There was a homogeneous distribution of students in terms of their current study semester.

**Table 2:** Sample characteristics (*n* = 37).

	**μ or *n***	**(*SD*) or %**
Gender (female)	30	81%
Age (*n* years)^*^	26.65	(5.21)
Year		
1st	24	65%
2nd	13	35%
*n* intend to enter a mental health profession		
Yes	27	73%
No	3	8%
Unsure	7	19%
Of those who said ‘*Yes'* (n = 27), *n* intend to enter…		
Clinical Psychology/Psychotherapy	23	85%
Counseling/Coaching	2	7%
Social Work	1	4%
Other: Neuropsychology	1	4%

μ, average value; n, count; SD, standard deviation; %, percentage.

* *Items, for which μ and SD were calculated*.

### Participants' Opinions About, and Familiarity, With AI/ML

The vast majority of respondents (36 of 37, 97%) had heard of “machine learning” and were familiar with “big data analytics” (29 of 37, 78%) (see Table 3). Respondents reported an average (mean) of 6.18 h, so far, of AI/ML in their degree. They anticipated, on average (mean), a further 12.43 h of AI/ML education in their Masters' degree program. Almost half (46%) of surveyed participants reported their intention to learn more about AI/ML as it pertains to mental healthcare, the remaining respondents were either unsure (43%) or responded that they had no intention of doing so (11%).

**Table 3:** AI/ML education experience and interest.

	***m* or *n***	**(*SD*) or %**	**Range**
*n* have heard of machine learning	36	97%	–
*n* are familiar with big data analytics	29	78%	–
*n* have read AI/ML mental health journal articles	23	62%	–
AI/ML education during the degree (*n* h)^*^			
So far	6.18	(16.63)	0 – 100
Predicted	12.43	(16.39)	0 – 60
Intend to learn about AI/ML as it pertains to mental health care			
Yes	17	46%	-
No	4	11%	-
Unsure	16	43%	-
Discussion about AI/ML should be part of clinical psychology education.^**^	4 (Moderately agree)	(1.48)	

m, mean; n, count; SD, standard deviation; %, percentage.

* Items, for which m and SD were calculated.

** *Answer was a rating on a five-point agreement Likert scale, where 1) Strongly disagree, 2) Moderately disagree, 3) Neutral, 4) Moderately agree and 5) Strongly agree*.

Students who intended to learn more about the application of AI/ML in mental health reported more hours of relevant education (m = 9.24) than those who were uncertain (m = 4.44). Furthermore, students who intended to learn more stated that they will have more hours of such education in the future (m = 20.88) compared with those who were unsure (m = 6.53). Using a five-point agreement Likert scale, where *1) Strongly disagree, 2) Moderately disagree, 3) Neutral, 4) Moderately agree and 5) Strongly agree* students moderately agreed” that discussions about artificial intelligence/machine learning should be part of clinical psychology/psychotherapy education.

The only significant positive relationship was between respondents' attitudes about the inclusion of AI/ML in education, and hours spent receiving relevant education. Students who reported receiving more hours of AI/ML education gave a higher rating on the five-point Likert scale (r = 0.34, p = 0.038).

### Results of Qualitative Findings

**Figure 1:**
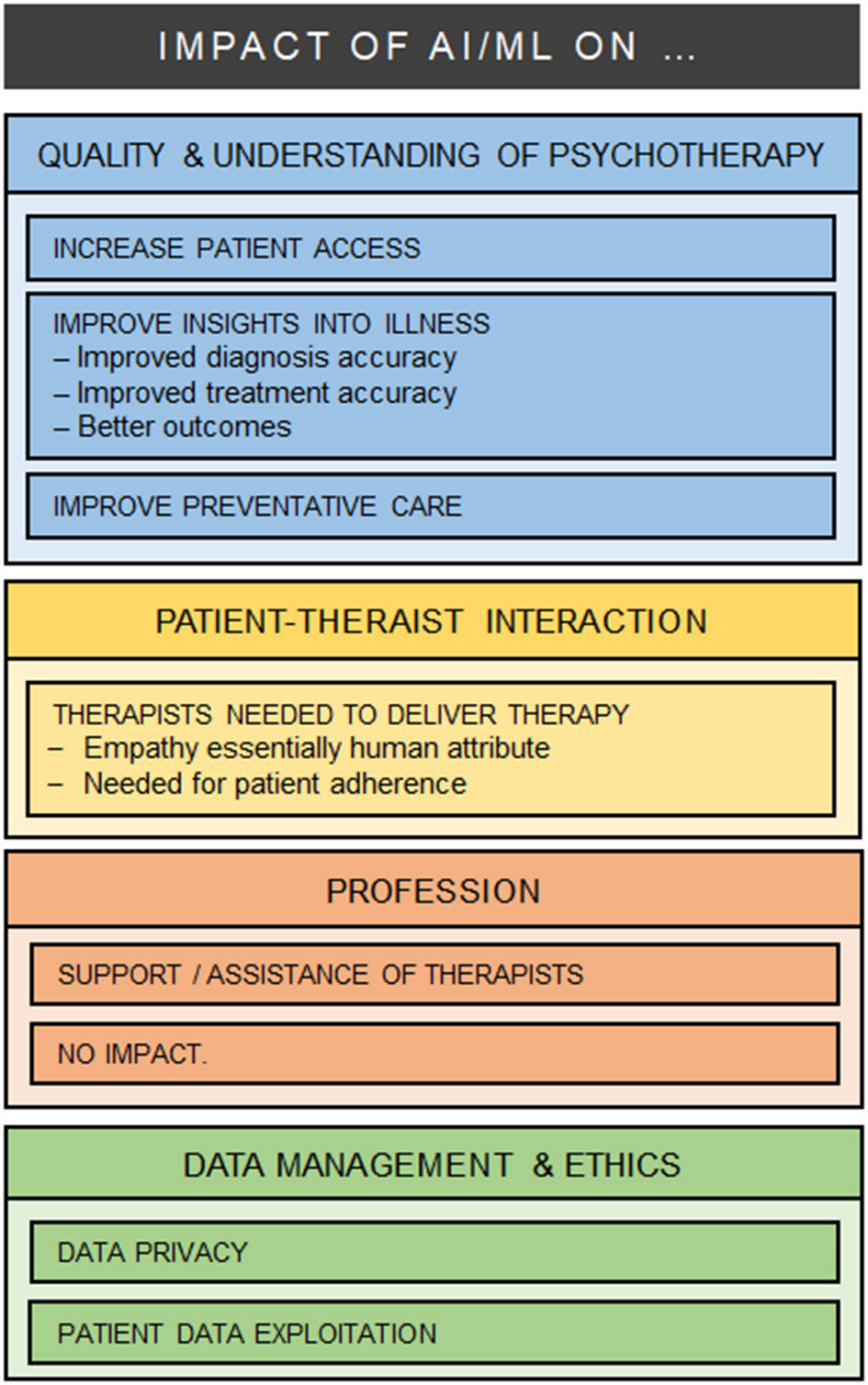
Themes and Sub-Themes.

All 37 participants responded to the open questions, and left comments (544 words) which were typically brief (one phrase or one or two sentences). As a result of the iterative analysis, four major categories were identified in relation to the impact of AI/ML on mental health care: (1) Changes in the quality and understanding of psychotherapy care; (2) Impact on patient-therapist interactions; (3) Impact on the psychotherapy profession; (4) Data management and ethical issues (see Figure 1).

#### Changes in the Quality and Understanding of Psychotherapy Care

Many comments reflected the view that AI/ML could facilitate and expand access to psychotherapy care and that this development would have a broad influence on public health; for example:

*Facilitating access to mental health services for example by providing online psychotherapy programs* [Participant 08]

*Machine learning can improve mental health care by adding more “knowledge”* [Participant 04]

Relatedly, many students believed that AI/ML will foster “*new patterns*” and research insights into mechanisms, and causes of mental illness; for example:

*There could be a shift from treating disorders to treating symptom clusters/categories* [Participant 14]

Massively more data can be gained and analyzed which could lead to completely new insights into underlying mechanisms of mental health

[Participant 17] Describing other benefits, some participants also predicted that AI/ML could lead to more accurate diagnoses, “*better outcomes*”, and more targeted treatments; for example,

*ML/AI may develop to help us in guiding our decision making in various health care situations, where a lot of information has to be taken into account and humans often lack to keep in mind all different outcomes or combinations (e.g., taking into account genetic variations, risk and protective factors, etc.)* [Participant 10]

*Personalize the prescription of psychopharmacological drugs, specifically decide on which SSRI is the best suited for a patient* [Participant 15]

Preventative care, risk detection, and “closer monitoring” also received a considerable number of comments; for example:

*AI will play a big role in the prevention of mental health conditions* [Participant 08]

*I think in general AI may help detect people struggling with mental health disorders that would have otherwise not be detected* [Participant 08]

*May become an additional guidance for treatment progression and predicting outcomes or identify high-risk patients*. [Participant 15]

However, not all participants agreed there might be broad benefits. Some students stressed that AI/ML-tools may be inaccurate or that the algorithms that power them are not humanly understandable. Others worried that innovations might induce a false sense of ease to access and get benefit from care; for example,

*Not clear how the algorithms work and how categorization takes place*. [Participant 14]

*I fear that patients will believe treatments via AI or machines will be easier and quicker and give them relief without much work* [Participant 03]

*Every individual is different and I personally am not a bit fan of too much digitalization, so perhaps these algorithms and parameters are suitable for the majority of the population, but not the minority* [Participant 11]

#### Impact on Patient-Therapist Interactions

Respondents frequently commented on the potential consequences of AI/ML for the patient-clinician relationship. Many respondents emphasized that a core feature of psychotherapy is the patient-clinician relationship, and that therapists would always be necessary to deliver care:

*I do not believe that machines will replace us as the client-therapist relationship is crucial to therapy*. [Participant 17]

*People need people. Artificial intelligence/machine learning should give us more time to spend with other people not replace relationships*. [Participant 35]

*As the current Covid-19 experience shows: many people are not happy with online therapy for a longer period of time since they miss the personal exchange with the therapist in the room (i.e. not in their own home nothing special anymore: like another business meeting on Zoom)*. [Participant 26]

*But I think the key in psychotherapy is the relationship between the patient and therapist. [It's] a work I think [which] is only effective when patients feel and experience real contact to the therapist a human being. I cannot imagine that AI can substitute us rather I think [it'll] be a support-tool for us*. [Participant19]

Multiple comments reflected concern that therapy depends on human attributes such as empathy (e.g., “*Computer can't give you empathy”*), and warmth (e.g., “*the social interaction warmth and real relationship will still be important”*). While a few participants suggested AI/ML might reduce the barriers to treatment participation and increased adherence, other comments proposed that human therapists would be necessary to ensure patient motivation and treatment adherence; for example:

*Not sure how motivating apps can be when you know it is just an app and not a real person expecting you to do tasks etc*. [Participant 07]

*[…] in the end they [the patients] will realize that it will not help them long-term, which could cause them even more suffering* [Participant 03]

*If used too often, patient could feel abandoned and put away with a robot*. [Participant 16]

Notably, only one student foresaw positive scope for AI/ML in traditional patient-therapist relationship:

“*A lot of therapists vary in many ways. And they make human mistakes a machine (virtual therapist) is less likely to have a bad day or feel [antipathy] for the patient. people can build a good relationship to a virtual therapist as well as long as they feel understood and accepted.”* [Participant 24]

Finally, while the patient-clinician interaction was widely commented upon, consideration of particular patient populations was rare; one participant suggested that “*Patients (for example older patients) could hesitate about doing a test since they might not trust the AI.”* [Participant 30]

#### Impact on the psychotherapy profession

Respondents' comments encompassed a number of predictions on the impact of AI/ML on therapists. Most respondents expressed the view that AI/ML-enabled tools will provide new ways to support, complement or assist therapists in carrying out their tasks; for example:

*It can be a helpful tool, to complement the therapeutic work*. [Participant 16].

*We can use AI as a tool to support our work*. [Participant 19]

*Internet-based treatments as a “homework” for patients could facilitate the change process in order to make it clearer for patients what the psychologist is trying to communicate*. [Participant 33]

Numerous comments highlighted positive benefits to therapists of AI/ML tools with respect to more basic and routine tasks, or in delivering care for patients with less serious psychological problems; for example:

*AI could […] also be performing standardized tests with mental health patients and the results of these tests would be shown directly to the clinical psychologist and psychotherapist. Based on those results, the AI might also conclude what the next goal in the psychotherapy with mental health patients would be and therefore aid the clinical psychologist and psychotherapists to look what the next step is for the patient*. [Participant 30]

*Minor issues will be treated via AI. Like chatbots. With minor issues I [mean] every day struggles or small psychological issues like a stressful life phase. AI will not take care of bigger problems and issues*. [Participant 16]

Some participants suggested that AI/ML-tools would help to relieve therapists of some workplace burdens, allowing them to devote more time to other important aspects of care by leaving the execution of bureaucratic work to technology; for example:

*Would help clinicians to speed up a lengthy process*. [Participant 18]

*I rather believe that it would help psychologists to have enough time in order to build good relationships with their clients*. [Participant 38]

*[It will allow more focus on] what therapists really excel at, maybe bureaucratic work could be cut down with AI/ML*. [Participant 23]

Perhaps with this in mind, some comments emphasized the possible impact that AI/ML might have both for the education and the training of therapists; for example:

*They will use more often computers and programs. Need to know more about programming and other technical knowledge*. [Participant 09]

*Practitioners might need to learn to apply certain AI/ML applications that have been shown to help improve decision making*. [Participant 10]

*I think clinical psychologists and psychotherapists will have to use artificial intelligence/machine learning. And therefore, have a certain knowhow in doing so* [Participant 35]

Finally, a number of participants expressed the view that AI/ML will have no significant impact on therapists in the short or long term; for example:

*My job would probably stay similar*. [Participant 07]

*I don't think related to this subject too much will change in 25 years*. [Participant 37]

*I think that the job of clinical psychologists and psychotherapists won't change that much*. [Participant 20]

*Artificial intelligence/machine learning can't replace psychotherapist/mental health professionals*. [Participant 09]

#### Data Management and Ethical Issues

Comments frequently described the “*massive amounts of data*” that can be accumulated through AI/ML-enabled tools, and many students expressed considerable concern about “*infringement of privacy*” with respect to data curation; for example:

*How securely are the data stored?* [Participant 07]

*To create trust, a transparent and secure way of data storage and protection would have to be provided*. [Participant 05]

*Of course, security and data protection are a crucial issue in the field of AI especially when it comes to sensitive information like mental health*. [Participant 08]

*Data security is probably the most important concern, where we really do lack the infrastructure for safe data collection and processing*. [Participant 23]

In respect of this, several students also identified the possibility of patient data exploitation as a problem; for example:

*Who will have access to the personal information of patients?* [Participant 24]

*Who will misuse them for commercial reasons… or will health insurance be able to track the patients' digital footprints – data protection?* [Participant 24]

*Potential leakages of patient information on the internet to unwanted recipients or hackers. Thus, AI must have a tight security system, or it must automatically be able to recognize potential hazards and dangers*. [Participant 30]

Notably, some students raised broad ethical concerns about the impact of AI/ML on mental health care but did so in a vague or truncated manner; for example:

*Autonomy or ethical problems*. [Participant 19]

*The ethics are quite complex*. [Participant 15]

*Is it ethical to monitor patients[?]* [Participant 31]

## Discussion

### Summary of Major Findings

The opinions and experiences of trainee clinicians have been missing from the debate about the impact of AI/ML on clinical psychology and psychotherapy. This exploratory survey indicates that clinical psychology students express some awareness of AI/ML. Most postgraduate students in our sample intended to enter a mental health profession, and most had some familiarity with the terms “machine learning” and “big data.” Around two thirds of respondents also reported reading a journal article on AI/ML. Around half (46%) the respondents reported their intention to learn more about AI/ML; remaining respondents were unsure, and around one in 10 reported no intention of doing so. Respondents also reported receiving an average of 6.18 h learning, so far, on the topic of AI/ML in their course and expected an average of a further 12.43 h of teaching on the topic in their degree program. Combining both reported and anticipated time on AI/ML education, this amounts to a perceived total of 18.61/3,600 h, or 0.52% of their total degree.

In light of limited course instruction, students demonstrated a wide range of views and some knowledge about AI/ML tools in psychotherapy. Participants commonly expressed the belief that AI/ML tools will help to expand access to care. Students frequently described the possibility of improving diagnostic and treatment insights, and preventative mental healthcare. While the term “digital phenotyping” (10, 11) was not used, students recognized the widely discussed potential of digital devices to gather moment-by-moment data that may be relevant to mental diagnosis and symptom monitoring (12, 13).

Students were divided about the impact of AI/ML on the future of their profession, findings that replicate a tension observed in other clinician surveys (4, 7). In line with these survey findings of practicing clinicians, students were skeptical that digital tools could replace human therapists in the delivery of care and considered empathy to be a quintessentially human attribute. Many students similarly forecast that digital technologies would be restricted to a supporting role, augmenting the role of the therapist or in undertaking automation of more routine tasks, though the specification of these tasks was often vague or unmentioned.

However, unlike other surveys on the future of the clinical professions (5, 6) students frequently expressed their ethical concerns about the impact of AI/ML on healthcare, especially in relation to patient privacy and data exploitation. Indeed, loss of privacy, and misuse of sensitive healthcare information remains a risk, with known cases of mobile technologies selling patient data to third parties (14–16). Mental health patients remain among the most vulnerable of patient populations and are especially at risk of privacy violations via the exploitation of their data, and it was clear that many of the students had reflected on this problem.

On the other hand, ethical considerations such as the “digital divide” in healthcare, patient digital literacy in using apps, and problems associated with algorithmic biases in the design of digital health tools received little or no attention (14). In addition, the regulation, approval, and evidence-base associated with currently available mental health apps received scarce commentary. These omissions may be viewed as concerning. As the digital app economy continues to boom there is considerable promise, but also the potential for harm. To date, it is estimated that there are more than 10,000 health apps available for download, yet most have never been subject to robust standards of evidence-based medicine (2, 17). While there is considerable scope for mobile health innovations in improving patient care (18, 19), there is also a pressing need to formulate clear recommendations for these apps among patients and clinicians.

Despite expressed ethical worries, it was also notable that some students believed that AI/ML would have no impact on psychotherapy in the short or long term, and around half of those surveyed suggested that they were unsure, or would not, follow up with more learning on AI/ML. We might cautiously infer from this that students did not consider it relevant to their job to provide advice to patients about the benefits and risks of currently available psychotherapy apps, for example or symptom monitoring. Again, this emerged as a concern. These tensions, and omissions may reflect lack of formal training about how AI/ML is already encroaching on mental health care. In addition, it is possible that students' current familiarity may be driven less by formal education than by outside sources, including the media.

Reflecting on these findings, the important question arises about whether teaching bodies and curricula should be adapted, not only for students but also for educators. In a recent survey, leading healthcare informaticians forecast that by 2029, AI/ML will incur workplace changes in primary care, with the need for increased training requirements in these fields (20). The present survey therefore raises questions about the preparedness of clinical psychology/psychotherapy students to fully engage in pressing debates about ethical and evidence-based issues pertaining to AI/ML tools, and in guiding patients on the use of psychotherapy and other mental health apps (21).

### Strengths and Limitations

To our knowledge, this pilot survey is the first to investigate the exposure, and opinions of, clinical psychology/psychotherapy students to AI/ML. The average response rate for online surveys is 20–30% (22). While our response rate achieved 31%, the overall sample size was small. The survey was administered during the COVID-19 pandemic and this may have affected willingness to respond. Relatedly, it is not known how, or whether, contextual conditions influenced their responses to the survey. With the recent uptick in telemedicine, and considerable debate about digital health during the pandemic, it is conceivable that participants' answers may have been influenced by both global and local conditions. Response biases might also have affected findings: a high number of our participants (23 of 37, 62%) reported having read AI/ML mental health journal articles. It is unknown, however, whether the decision to complete the survey was influenced by students' prior knowledge or awareness of the topic of AI/ML. The convenience sample of students at a single academic center, also raises questions about representativeness.

Some items on the survey could be challenged on the grounds of vagueness. For example, “familiarity with big data analytics” might, justifiably, be considered semantically opaque. While we acknowledge that this survey item is coarse-grained, this preliminary study set out to explore general student awareness, level of personal inquiry, and formal educational exposure to the topic of AI/ML. We recommend that interviews, or focus groups would provide finer-grained analysis of student awareness and opinions of AI/ML. Further, we suggest that future research might usefully explore the views of specific groups of students (for example, only those who aim to work as psychotherapists), and on the views of clinical psychology/psychotherapy, and other mental health educators. In addition, it would be useful to evaluate course curricula across tertiary level colleges and universities to obtain a more objective assessment of topics and level of education about AI/ML in clinical psychology/psychotherapy training.

## Conclusions

Clinical psychologists and psychotherapists entering the job-market will face new challenges posed by the emergence of new e-health tools based on artificial intelligence, machine learning and big data analytics. Although the majority of students in our survey had heard of “machine learning” and read about AI/ML in journal articles, only half of respondents planned to learn more about AI/ML as they pertain to mental health care. Importantly, most students agreed that discussions about AI/ML should be part of clinical psychology/psychotherapy education. Yet they estimated only 0.52% of their total degree (18.61/3,600 h) will be dedicated to these topics.These results seem to contrast with current trends. Clinical psychologists/psychotherapists—as well as patients/clients—can already access thousands of digital tools, online services and mobile apps based on AI/ML that have been specifically designed to integrate or substitute traditional mental healthcare services or consultations. The impact of these technologies on mental healthcare is set to rise as new and more advanced AI/ML tools and services are released.

We suggest that clinical psychology/psychotherapy curricula should embrace these new challenges in educating the clinicians of tomorrow. Courses might be usefully designed to train clinical psychologists and psychotherapists on how to guide and assist patents in being “digitally savvy” — and in making informed choices about available AI/ML tools and services. With this in mind, we envisage a need for interdisciplinary approaches to psychotherapy education. For example, psychotherapists with computer/informatics backgrounds, and psychotherapy ethicists with training on digital healthcare should devise relevant short courses for students and continuing professional development.Course curricula should encompass instruction on when patients might benefit from using apps, and/or when they should consult with therapists, in-person. Courses should therefore encompass discussion about the evidence-based effectiveness and safety of mental health apps, as well as about other delicate ethical and regulatory issues related to privacy, equality, and discrimination. Psychotherapy practitioners and students should feel empowered to keep abreast of new technological advances including what these developments mean for their profession and their patients.

## Data Availability Statement

Upon request, the raw data supporting the conclusions of this article will be made available by the authors, without undue reservation.

## Ethics Statement

The studies involving human participants were reviewed and approved by University of Basel. The patients/participants provided their written informed consent to participate in this study.

## Author Contributions

CL, JG, MA, AK, and CB: administered survey, collected data, and revised the manuscript. AK, MA, and CL: data analysis. CB, CL, AK, and MA: wrote the first draft of paper. All authors contributed to the article and approved the submitted version.

## Conflict of Interest

The authors declare that the research was conducted in the absence of any commercial or financial relationships that could be construed as a potential conflict of interest.
