# Supplementary material for: Machine Learning in Clinical Psychology and Psychotherapy Education: A Mixed Methods Pilot Survey of Postgraduate Students at a Swiss University
Source: Front Public Health. 2021 Apr 9;9:623088. doi: 10.3389/fpubh.2021.623088 (PMC8064116; doi:10.3389/fpubh.2021.623088)
Supplement: Supplementary file 1 [file Data_Sheet_1.PDF]

MACHINE LEARNING AND THE FUTURE OF MENTAL HEALTH CARE:

OPINIONS OF PSYCHOLOGY STUDENTS AT A SWISS UNIVERSITY

Dear Student,

Researchers at the Division of Clinical Psychology and Psychotherapy, University of Basel invite you to take part in a survey. We are inviting you, as psychology students to give your opinions about technology and the future of mental health care. This research will help to inform clinical psychology educationalists and policy-makers.

The online survey should take around 10 (and no more than 15) minutes to complete. We will not collect any identifying information from you, and your responses will be pseudonymized. Your response will be collated with those of other respondents in aggregated, pseudonymized form. **This survey is not a test; we are interested in your opinions as clinical psychology and psychotherapy students.** We refer you to the Information sheet and consent form for more information.

If you decide to participate, we appreciate your time and contribution to our research.

Thank you.

Dr Cosima Locher, University of Basel

Email contact: cosima.locher@unibas.ch

SECTION A

In this section we will ask demographic questions.

A1. Gender. Please select box.

Male ☐ 1

Female ☐ 2

Other ☐ 3

No information ☐ 4

A2. Year of birth. Please enter year. ☐

A3. Current semester of your Masters degree. Please select box.

1st ☐ 1

2nd ☐ 2

3rd ☐ 3

4th ☐ 4

A4. What was your undergraduate degree subject(s)? Please enter.

☐

A5. Do you intend to enter a mental health profession?

Yes ☐ 1 → **If answered "Yes" please go to Question A6 first**

No ☐ 2 → **If answered "No" or "Unsure" please go to Question A7**

Unsure ☐ 3

A6. Please select one box only.

Clinical Psychology/Psychotherapy ☐ 1

Social work ☐ 2

Counselling/Coach ☐ 3

Other (please specify) ☐ 4

Thank you for completing Section A.

Now turn to Section B.

SECTION B

The questions in this section are on your opinions about the impact of machine learning/artificial intelligence on the future of mental health care. We do not assume you have any expertise about machine learning or artificial intelligence in health care.

B1. In the next 25 years, please briefly describe the way(s) you believe artificial intelligence/machine learning might change the care of patients with mental health conditions.

B2. In the next 25 years, please briefly describe the way(s) you believe artificial intelligence/machine learning might change the job of clinical psychologists and psychotherapists.

B3. Please provide any brief comments you may have about the <u>potential benefits</u> of artificial intelligence/machine learning to the care of patients with mental health conditions.

B4. Please provide any brief comments you may have about the <u>potential risks</u> of artificial intelligence/machine learning in the care of patients with mental health conditions.

Thank you for completing Section B.

Please turn your attention to Section C.

SECTION C

The questions in this section ask about your familiarity with artificial intelligence/machine learning.

C1. Have you heard of machine learning?

No ☐ 1

Yes ☐ 2

C2. Are you familiar with big data analytics?

No ☐ 1

Yes ☐ 2

C3. Have you read any academic journal articles about artificial intelligence/machine learning in mental health care either during your studies or by yourself?

No ☐ 1

Yes ☐ 2

C4. Please estimate how many hours your instructors/lecturers *<u>have spent</u>* discussing artificial intelligence/machine learning during your Masters degree so far.

☐

C5. Please estimate how many hours your instructors/lecturers *<u>will spend</u>* discussing artificial intelligence/machine learning during the course of obtaining your Masters degree.

C6. Do you plan to learn about artificial intelligence/machine learning as they pertain to mental health care?

No ☐ 1

Yes ☐ 2

Maybe ☐ 3

C7. Discussion about artificial intelligence/machine learning should be part of clinical psychology/psychotherapy education.

Strongly disagree ☐ 1

Moderately disagree ☐ 2

Somewhat disagree ☐ 3

Somewhat agree ☐ 4

Moderately agree ☐ 5

Strongly agree ☐ 6

Thank you for completing Section C

THANK YOU FOR TAKING THE TIME TO COMPLETE THIS SURVEY
